# Supplementary material for: Depression and associated factors among older people in Vietnam: Findings from a National Aging Survey
Source: PLoS One. 2024 May 10;19(5):e0299791. doi: 10.1371/journal.pone.0299791 (PMC11086836; doi:10.1371/journal.pone.0299791)
Supplement: Attachment — Submitted filename: Response to Reviewers.docx [file pone.0299791.s002.docx]

**Response to Reviewers and Editor for the manuscript “Depression and associated factors among older people in Vietnam: findings from a National Aging Survey”**

Dear PLOS ONE Editor, Reviewers,

We would like to express our gratitude to the editor and reviewers for the feedback and helpful comments to improve our manuscript.

We have made all attempts to fully address the editor and reviewers’ comments in the revised manuscript. We believe the additional revisions based on reviewers’ comments have helped to substantially improve our manuscript. The revised manuscript has been submitted alongside this response to the reviewer’s letter.

Below, we have outlined how we have handled the Editor’s additional requirements and reviewers’ comments. We have highlighted our responses to each comment in bold.

**Responses to Journal Requirements**

When submitting your revision, we need you to address these additional requirements.

1. Please ensure that your manuscript meets PLOS ONE's style requirements, including those for file naming. The PLOS ONE style templates can be found at

<https://journals.plos.org/plosone/s/file?id=wjVg/PLOSOne_formatting_sample_main_body.pdf> and

<https://journals.plos.org/plosone/s/file?id=ba62/PLOSOne_formatting_sample_title_authors_affiliations.pdf>

**Response: We have revised and formatted the manuscript following PLOS ONE's style requirements.**

2. Thank you for stating the following in the Acknowledgments Section of your manuscript:

"The study utilized the data source from the Vietnam National Aging Survey (VNAS) conducted in 2022 by the Institute of Social Medical Studies and funded by the Asian Development Bank."

We note that you have provided additional information within the Acknowledgements Section that is not currently declared in your Funding Statement. Please note that funding information should not appear in the Acknowledgments section or other areas of your manuscript. We will only publish funding information present in the Funding Statement section of the online submission form.

Please remove any funding-related text from the manuscript and let us know how you would like to update your Funding Statement. Currently, your Funding Statement reads as follows:

"The authors received no specific funding for this work."

Please include your amended statements within your cover letter; we will change the online submission form on your behalf.

**Response:**

**We have deleted the statement about the funding of the Vietnam National Aging Survey (VNAS), which we analyzed data from for this paper in the Acknowledgement section. As we did not receive any funding for our work, we would like to keep our current Funding Statement "The authors received no specific funding for this work".**

3. We note that you have indicated that data from this study are available upon request. PLOS only allows data to be available upon request if there are legal or ethical restrictions on sharing data publicly. For more information on unacceptable data access restrictions, please see <http://journals.plos.org/plosone/s/data-availability#loc-unacceptable-data-access-restrictions>.

In your revised cover letter, please address the following prompts:

a) If there are ethical or legal restrictions on sharing a de-identified data set, please explain them in detail (e.g., data contain potentially sensitive information, data are owned by a third-party organization, etc.) and who has imposed them (e.g., an ethics committee). Please also provide contact information for a data access committee, ethics committee, or other institutional body to which data requests may be sent.

b) If there are no restrictions, please upload the minimal anonymized data set necessary to replicate your study findings as either Supporting Information files or to a stable, public repository and provide us with the relevant URLs, DOIs, or accession numbers. For a list of acceptable repositories, please see <http://journals.plos.org/plosone/s/data-availability#loc-recommended-repositories>.

We will update your Data Availability statement on your behalf to reflect the information you provide.

**Response: Following the journal request, we have made the data fully available.**

**We have uploaded a minimal anonymized data set as a Supporting Information file.**

Additional Editor Comments:

Please address the reviewers' comments, especially the concerns on the validity of GDS-15.

**Response: We have addressed all reviewers' comments, including the concerns about the validity of GDS-15.**

**Responses to Reviewers’ comments**

**Thank you very much for your valuable comments/suggestions. We have revised the manuscripts based on your comments/suggestions. Our responses to each of your comments are below.**

**We have highlighted our responses to each comment in bold.**

Comments to the Author

1. Is the manuscript technically sound, and do the data support the conclusions?

The manuscript must describe a technically sound piece of scientific research with data that supports the conclusions. Experiments must have been conducted rigorously, with appropriate controls, replication, and sample sizes. The conclusions must be drawn appropriately based on the data presented.

Reviewer #1: Partly

Reviewer #2: Yes

**Response: We have revised the method section and provided more details about the methodology following the reviewer’s comments. We hope that the revised manuscript is technically sound, and the data support the conclusions.**

2. Has the statistical analysis been performed appropriately and rigorously?

Reviewer #1: Yes

Reviewer #2: Yes

3. Have the authors made all data underlying the findings in their manuscript fully available?

The [PLOS Data policy](http://www.plosone.org/static/policies.action#sharing) requires authors to make all data underlying the findings described in their manuscript fully available without restriction, with rare exception (please refer to the Data Availability Statement in the manuscript PDF file). The data should be provided as part of the manuscript or its supporting information, or deposited to a public repository. For example, in addition to summary statistics, the data points behind means, medians and variance measures should be available. If there are restrictions on publicly sharing data—e.g. participant privacy or use of data from a third party—those must be specified.

Reviewer #1: No

Reviewer #2: Yes

**Response: Following the reviewer’s comment and journal request, we have made the data fully available.**

**We have submitted a data set as a Supporting Information file.**

4. Is the manuscript presented in an intelligible fashion and written in standard English?

PLOS ONE does not copyedit accepted manuscripts, so the language in submitted articles must be clear, correct, and unambiguous. Any typographical or grammatical errors should be corrected at revision, so please note any specific errors here.

Reviewer #1: No

Reviewer #2: Yes

**Response: We have done a thorough proofreading and have made grammar corrections for the revised manuscript.**

5. Review Comments to the Author

Please use the space provided to explain your answers to the questions above. You may also include additional comments for the author, including concerns about dual publication, research ethics, or publication ethics. (Please upload your review as an attachment if it exceeds 20,000 characters)

**Responses to reviewer # 1’s comments**

Reviewer #1: Dear Editor,
I appreciate the opportunity to review the manuscript titled 'Depression and Associated Factors Among Older People in Vietnam: Findings from a National Aging Survey.' This study utilizes the Vietnam National Aging Survey (VNAS) conducted in 2022 to assess the prevalence of depressive symptoms and associated factors among older people in Vietnam. The findings indicate a 20.2% prevalence of depressive symptoms among older people and identify various factors associated with depression. I would like to offer several suggestions to enhance the manuscript's quality.
Abstract:
1. Methods: The abstract lacks information about the 'factors' evaluated in the study. Adding a brief statement about these factors will enhance clarity for readers and help them understand what was assessed.

**Response: We have added a list of factors associated with depression that the study assessed in the abstract/methods (lines 38-41).**

Introduction:
2. Line 59: What is the current status of population aging in Vietnam? What percentage of the population is considered older, etc.?

**Response: We have added the information “The number of people aged 60 and over was 12.58 million which accounted for 12.8% of the total population in 2021” (Lines 58-59).**

3. Line 65: It appears that there are already previous studies (references 4-10) quantifying the burden of depression among the population of interest. Therefore, a robust justification for the current study is needed.

**Response: We have added a justification for the need of the current study. (Lines 91-95 in the revised manuscript).**

4. The authors offer a general justification, noting that prior research (references 4-10) relied on samples of moderate size and limited representativeness, and utilized scales that were not tailor-made for assessing depressive symptoms among the older adults. Enhancing the manuscript's clarity with details about the specific samples and scales employed in each of these studies would help describe these methodological constraints. For instance, it would be valuable to understand the characteristics of the samples used in those studies, the scales utilized, and how the present methodology addresses these limitations.

**Response: We have added a description with details about the specific samples and scales employed in each of the prior studies conducted in Vietnam. (Lines 78-90 in the revised manuscript).**

5. Line 78: Author provide general ration that previous studies (reference 4-10) employed a moderate or small and unrepresentative sample as well as use of scale specifically not designed to assess the depressive symptoms among older people. A description for each study on these methodological limitations will add clarity to evaluate their methodological limitations. For example, what samples and scales were used by each of these studies and how does use of current tool address those limitations?

**Response: We have added a description with details about the specific samples and scales employed in each of the prior studies. (Line 78-90 in the revised manuscript). We have also added a justification of advantages of the current study with a large and representative sample of older people and the employment of GDS-15, a depression screening scale specifically designed for older people. (Lines 91-95 in the revised manuscript).**

6. Line 84-86: These statements seem more like methodological details. Including this level of methodological detail here may make the following paragraph sound repetitive.

**Response: We have deleted this statement and revised the purpose of the study (Lines 95-96 in the revised manuscript).**

Methods:
7. Is the VNAS dataset publicly available?

**Response: Following the reviewers’ comment and journal request, we have made the data fully available.**

**We have submitted a data set as a Supporting Information file.**

8. Could you please explain the terms "ecological regions" and "communes" in the local context? Adding explanatory phrases for such terms, which may not be familiar to international readers, would be beneficial.

**Response: Vietnam is divided into 6 socio-economic regions. Commune is the lowest administrative unit.**

**We replaced the term ‘ecological’ with ‘socio-economic’ (Line 105). An explanatory phrase for the term “commune” was added (Line 108).**

9. Line 106: Please cite the 'Census and Survey Processing System,' the computer-assisted interview program.

**Response: We have added a reference for the 'Census and Survey Processing System (CSpro), the computer-assisted personal interview program, and have cited it (lines 120-121).**

10. Who collected the data, and how was the quality of data collection maintained? How many surveyors were involved?

**Response: Twenty-four persons who were last-year students or recent graduates from public health, sociology, or social science Universities were trained to be interviewers. Six field supervisors were responsible for quality control of the data collection. Field supervisors observed selected interviews for each interviewer to ensure that the interviewer followed data collection and interviewing procedures. The CSpro data entry form was built with interactive logic and consistency checks. The program would show data errors immediately on the tablet screen during the interview so the interviewer could check and correct the errors in real-time. Field supervisors checked all completed questionnaires at the end of the data collection day by running a data-checking program. Field supervisors then asked interviewers to correct errors and fill in any missing information.**

**Descriptions of data collectors and quality controls were added (line 121-130)**

11. What was the reliability of the GDS scale in the sample? Has the validity of the GDS been established in Vietnam? The same comment applies to other scales used in this study, such as IADL.

**Response: The Vietnamese version of GDS-15 was validated and used in several studies with older people in Vietnam. In this study, the internal consistency of GDS-15 was good with a Cronbach’s alpha of 0.79.**

**We have added this information with citations (lines 137-139).**

**The ADL and IADL were validated and used in several studies with older people in Vietnam. In this study, Cronbach’s alpha for the ADL was 0.89, and for the IADL was 0.9.**

**These information were added (lines 167-169, lines 176-178).**

12. How was the wealth index score calculated from the described variables? Was it a simple addition? How many items were included, and what was the methodology?

**Response: We used principal component analysis (PCA) to create a wealth index score from 8 questions about housing characteristics, household utilities, and household assets.**

**We have added this description in the method section (lines 149-152).**

13. On page 127, the authors mentioned, "In data analysis, the responses were grouped into: Very Poor/Poor, Fair, and Good/Excellent," but did not provide a rationale for combining these groups together.

**Response: Many previous studies grouped self-reported health status into 3 categories. To be comparable with previous studies we combined the self-reported health status responses into 3 categories: Very Poor/Poor, Fair, and Good/Very Good.**

**We have added this rationale with citations (lines 155-156).**

14. On page 128, how was the disease information captured? Was it based on self-report, medical records, or some lab measures? Please provide specific details about the questions asked or measurements taken.

**Response: The disease information was captured based on self-report.**

**We used one question asking if the respondent has ever been diagnosed with chronic diseases including Arthritis, Angina, Diabetes, Chronic Lung diseases (emphysema, bronchitis, Chronic obstructive pulmonary diseases), Blood pressure problems, Cancer, Kidney diseases, Heart diseases, Liver diseases.**

**We have added this information (lines 157-158).**

15. The measurement of variables like cigarette smoking and alcohol use is not clear and could benefit from a brief description of the specific questions asked.

**Response: We have added brief descriptions of the specific questions asking about cigarette smoking and alcohol use (lines 182-187).**

16. How was social support measured? Was it a single-item question, or were pre-validated tools used?

**Response: Social support was measured using 5 questions asking about living arrangements (e.g., living with children), number of family members, number of living children, receiving financial support for daily living in the last 12 months, and social participation in the last 12 months.**

**We have added this information in the measure section (lines 188-190).**

17. Line 160: The definition of depression is already specified in the dependent variable section, so mentioning it here again seems redundant.

**Response: We deleted this definition of depression in the data analysis section.**

18. Regarding data analysis, how were the normality of variables assessed, and was multicollinearity checked?

**Response:**

**We used logistic regressions to explore the associations between depression and other factors. Logistic regression does not require independent variables to follow a normal distribution, we did not need to check the normality of variables included in the regression models.**

**Here is one reference that discusses the normal distribution test for logistic regression and linear regression. “Logistic regression does not require residuals to follow a Normal distribution so testing for normality is not needed like it is in Linear regression”. (**[**https://www.researchgate.net/publication/341354759_Logistic_and_Linear_Regression_Assumptions_Violation_Recognition_and_Control**](https://www.researchgate.net/publication/341354759_Logistic_and_Linear_Regression_Assumptions_Violation_Recognition_and_Control)**)**

**We used the correlation matrix, tolerance, and VIF criteria to check multicollinearity for the variables included in the logistic regression analyses.**

**We have added this statement “Multicollinearity was checked using Tolerance and VIF criteria to ensure that the independent variables included in the logistic regression models were not highly correlated with each other” (lines 201-202).**

19. Which variables were adjusted in the model?

**Response:**

**We included all variables with a P-value <0.2 in the bivariate analyses in the logistic regression model. The association of the dependent variable and the independent variable of interest in the model was adjusted for all other independent variables included in the model.**

**We have added a note for Table 2 “All variables with a P-value <0.2 in the bivariate analyses were included in the logistic regression models.”**

**We have added a citation/reference for the use of p-value <0.2 to select variables from bivariate analyses to include in the logistic regression models in the data analysis section (Line 200).**

Discussion:
20. What are the strengths of this study?

**Response: We discuss the strength of this study in the discussion (lines 292-298)**

21. What are the implications of the findings for public health practice and policy? Please provide specific recommendations based on your findings.

**Response: We have added specific recommendations based on the findings in the conclusion section (Lines 363-374).**

Tables:
22. Please specify whether the OR in Table 2 is adjusted or unadjusted. Also, mention in the footnote the variables that were adjusted for.

**Response:**

**The OR is adjusted. ‘Adjusted’ was added to the OR.**

**We included all variables with a P-value <0.2 in the bivariate analyses in the logistic regression model. The association of the dependent variable and the independent variable of interest in the model was adjusted for all other independent variables included in the model.**

**We have added a note for Table 2 “All variables with a P-value <0.2 in the bivariate analyses were included in the logistic regression models.”**

**We have added a citation/reference for the use of p-value <0.2 to select variables from bivariate analyses to include in the logistic regression models in the data analysis section (Line 200).**

Overall:
23. Grammar errors are prevalent in several parts of the manuscript, indicating the need for substantial editing and thorough proofreading to rectify grammar-related issues.

**Response: We have done a thorough proofreading and have made grammar corrections for the revised manuscript.**

**Thank you very much for your comments/suggestions.**

**Responses to Reviewer #2’s comments**

Reviewer #2: This study has examined the prevalence and risk factors of depressive symptoms among older adults in Vietnam
The strength of the study is the use of nationally representative data and the results suggest the role of chronic diseases and functional limitations on depressive symptoms. Several studies have identified the similar results across the globe.

1. In line number 106, you can mention it as Computer-assisted personal interviewing (CAPI) instead of computer assisted interview program

**Response: We have revised the name of the program to “Computer-assisted personal interviewing (CAPI)” (lines 120-121).**

2. What is the response rate of the survey?

**Response: The response rate of the survey was 97.3%. We have added this in the method section (line 115).**

3. Validity of the 15-item Geriatric Depression Scale in Vietnam?, add the Cronbach’s alpha value

**Response: The Vietnamese version of GDS-15 was validated and used in several studies with older people in Vietnam. In this study, the internal consistency of GDS-15 was good with a Cronbach’s alpha of 0.79.**

**We have added this information and citations (lines 137-139).**

4. Whether the questions were translated in local language?

**Response: Yes, we used the Vietnamese version of the questionnaire to interview older people. The term ‘Vietnamese” was added to the “questionnaire” (line 119).**

5. You have included depression as a chronic and independent variable, there may some collinearity, therefore remove depression from the list of chronic diseases included as an independent variable

**Response: We removed depression from the list of chronic diseases. “Depression” was deleted from the list of chronic diseases in the measure section (lines 159-161).**

**The bivariate and multivariate analyses were rerun with the chronic diseases independent variable excluding depression. The results in Table 2 were revised** **accordingly.**

6. In table 1, the prevalence of chronic diseases were not included except depression and pls remove depression. The list of variable included in the table 2 must be available in table 1 also

**Response: We have added all variables presented in Table 2 in Table 1. Table 1 now includes all variables.**

**We have added descriptions of the newly added variables in Table 1 in the result section – characteristics of older people (Lines 213-219).**

**We have removed depression from the list of chronic diseases.**

**Thank you very much for your comments/suggestions.**
